# Preformed Fibril Seeding Reshapes the Phosphorylated α-Synuclein Proximal Proteome in the Olfactory Bulb

**DOI:** 10.64898/2026.07.22.740026

**Published:** 2026-07-27

**Authors:** Solji G. Choi, Atousa Bahrami, Jayda B Duvernay, Tyler Tittle, Ronald Melki, Jeffrey H. Kordower, Bryan A. Killinger

## Abstract

**Background:**

Aggregated alpha-synuclein (αsyn) phosphorylated at serine 129 (PS129) accumulates in synucleinopathies, with the olfactory bulb (OB) being severely affected. Non-aggregated physiological PS129 is abundant in the mammalian OB, where it likely modulates αsyn-protein interactions. The impact of aggregation on the PS129 interactome in the OB remains unclear. We hypothesized that αsyn aggregation alters the PS129 interactome, shifting canonical synaptic partners (e.g., SNARE proteins) toward the aggregate-associated network. To test this hypothesis , we mapped PS129 interactions in the OB of PFF-injected WT and SNCA ^A53T/A53T^ (M83) mice using biotinylation by antibody recognition (BAR) and pretreated with calf-intestine alkaline phosphatase (CIAP) to distinguish physiological from aggregate-associated PS129 interactomes. Seeding and spread were assessed by immunohistochemistry and in situ seeding immunodetection assay (isSID).

**Results:**

Following OB-PFF injections, CIAP-resistant aggregates and seeds were detected throughout the neuroaxis of M83 mice (e.g., OB to brainstem) but less so in WT mice. isSID seeding was concentrated near CIAP-resistant aggregates, but the overlap was only partial. BAR-PS129 identified 2,309 proteins in M83 OBs and 990 proteins in WT OBs. Of these, 357 proteins in M83 mice and 247 proteins in WT mice were associated with the CIAP-resistant, aggregate-enriched PS129 fraction. In both models, the CIAP-resistant interactome largely overlapped with the broader PS129 interactome, suggesting that seeded aggregation primarily affects existing PS129 interactions rather than directing the formation of new pathological ones. A conserved 107-protein CIAP-resistant signature shared between M83, and WT mice was enriched for axon–glia adhesion, myelin-associated, axonal/cytoskeletal, proteostatic, and synaptic vesicle-related proteins.

**Conclusion:**

Aggregated PS129 engages a subset of PS129 networks enriched at axon–glial interfaces. CIAP-resistant aggregates were partially associated with seed competency, indicating that CIAP resistance and seed competency are related but not equivalent. These results provide molecular details of αsyn seeding and spread from the OB.

## Full Text Availability

The license terms selected by the author(s) for this preprint version do not permit archiving in PMC. The full text is available from the preprint server.

